# Editorial: Zebrafish Epigenetics

**DOI:** 10.3389/fcell.2022.977398

**Published:** 2022-08-09

**Authors:** Vincenzo Cavalieri, Katie L. Kathrein

**Affiliations:** ^1^ Laboratory of Molecular Biology, Department of Biological, Chemical and Pharmaceutical Sciences and Technologies (STEBICEF), University of Palermo, Palermo, Italy; ^2^ Zebrafish Laboratory, Advanced Technologies Network (ATeN) Center, University of Palermo, Palermo, Italy; ^3^ Department of Biological Sciences, University of South Carolina, Columbia, SC, United States

**Keywords:** zebrafish, epigenetics, chromatin dynamics, histone post translational modifications, DNA methylation

A key area of focus in the field of epigenetics pertains the comprehension of the functional relevance of the epigenetic mechanisms occurring during embryogenesis to shape normal developmental trajectories and adult phenotypes (Atlasi and Stunnenberg, 2017; Skvortsova et al., 2018; Cavalieri, 2021; Marchione et al., 2021).

Several lines of evidence highlighted that the small freshwater cyprinid *Danio rerio*, commonly known as zebrafish, is an excellent vertebrate model for research purposes in the field of epigenetics (Huang et al., 2013; Balasubramanian et al., 2019; Horsfield, 2019; Cavalieri, 2020). The general strengths of zebrafish over concurrent models are well known: ease of husbandry and maintenance in laboratory, high fecundity, external fertilization, short life cycle and generation time. Beyond this, the increasing popularity of zebrafish for epigenetic research purposes is due to two main reasons. First, components of the epigenetic machinery have been widely characterized in zebrafish, showing overall conservation with mammals (Howe et al., 2013; Cavalieri and Spinelli, 2017). No less important, zebrafish embryos are optically translucent and relatively permeable to a wide range of compounds, allowing non-invasive live imaging of morphogenesis and phenotypes following exposure to environmental stressors that challenge the epigenome (Godinho, 2011; Ali et al., 2014). Altogether, these benefits also make zebrafish an outstanding model for large-scale screening of potential therapeutics targeting epigenetic regulatory mechanisms.

This Research Topic includes 7 original and review articles from 57 authors, covering key aspects of zebrafish research aimed at improving our understanding of the role of epigenetic mechanisms in biological processes occurring in zebrafish. For instance, Li et al. highlighted a positive correlation between neural induction aberrations and overexpression of the histone demethylases Kdm5a, Kdm5d, and Kdm7a in zebrafish morphants lacking the PRC1 member Pcgf1. They also observed decreased levels of both the repressive H3K27me3 and activating H3K4me3 marks at the promoter of pluripotent and neural markers, respectively, revealing that Pcgf1 might function as both a facilitator for pluripotent maintenance and a repressor for neural induction.

Similarly, DiNapoli et al. revealed the critical role of histone methylation in tumorigenesis using a transgenic zebrafish melanoma model expressing H3 lysine-to-methionine mutations at lysine 9 and 27. Using this system, they found that loss of H3K9 methylation suppressed melanoma formation, while loss of H3K27 methylation significantly accelerated melanoma formation. These findings highlight the power in understanding how specific epigenetic marks and their associated regulators play a significant role in tumor progression.

Another paper by Calvird et al. provided compelling evidence demonstrating that, following integration in the zebrafish genome, a large concatemeric promoter-reporter transgene recapitulates molecular hallmarks of heterochromatic silencing, including enrichment in H3K9me3, and that expression from the transgene array can be reactivated by depletion of known regulators of heterochromatin. These results provide a valuable tool for rapidly monitoring heterochromatin-mediated repression of repeats in a living vertebrate organism.

Strictly connected with all these facets is the work investigating how environmental cues trigger reversible remodelling of embryonic epigenetic configurations and phenotype alterations that can be inherited over multiple generations. In this regard, Terrazas-Salgado et al. provided a useful synopsis of the current knowledge regarding the advantages of using zebrafish as an experimental animal model to study the transgenerational effect of xenobiotics on key epigenetic processes, including DNA methylation, histone post-translational modifications, and noncoding RNA transcription.

Several evidence showed that key components of the epigenetic machinery specifically control substrates other than chromatin. In this connection, Łysyganicz et al. reported hypoacetylation/hyperglycylation of ciliary tubulin in distinct cell types of developing *hdac6* and *sirt2* mutant zebrafish, suggesting that Hdac6 and Sirt2 deacetylases probably regulate the activity of the tubulin acetylase enzyme(s).

Recently, comparative studies among different organs in different vertebrate species showed that alteration of gene expression profiles is strictly associated with alteration of the epigenetic landscape. Following this line of thought, Komada and Nishimura reviewed published studies associating microglial neuroinflammation with dysregulation of the epigenetic landscape, including changes in DNA methylation and miRNA transcription, using a comparative approach between zebrafish and mammal models.

Finally, in their review article, Panara et al. examined how the combination of the latest technologies in epigenetics, such as ATAC-seq, CAGE-seq, ChIP-seq, CUT&RUN and CUT&Tag, are being used in zebrafish to determine chromatin states and cis-regulatory elements that shape the zebrafish vascular network.

In conclusion, the collection of high-quality articles included in this Research Topic encompasses different relevant areas of investigation, demonstrating the impact that the zebrafish model has on our understanding of the interplay among epigenetic regulation dynamics and diverse biological processes. From these papers, it is evident that the field is highly active and producing exceptional work that warrants further research.

We would like to thank all authors, co-authors, and reviewers who accepted our invitation to contribute to this Research Topic, and we hope that the data and information we conveyed will be beneficial to the scientific community.
